# Identification of a novel HLA-G allele, HLA-G*01:66, using PolyseqOne and oxford nanopore sequencing

**DOI:** 10.3389/fgene.2026.1786514

**Published:** 2026-03-04

**Authors:** Ran Li, Liyan Sun, Siqi Cai

**Affiliations:** 1 Department of Laboratory Medicine, Shenzhen Blood Center, Shenzhen, China; 2 Institute of Transfusion Medicine, Shenzhen Blood Center, Shenzhen, China; 3 Department of Transfusion Medicine, The Eighth Affiliated Hospital of Sun Yat-sen University, Shenzhen, China

**Keywords:** codon, HLA-G, mutation, nanopore sequencing, novel allele

## Abstract

HLA-G*01:66 differs from HLA-G*01:01:22:01 by one missense nucleotide substitution at position 508 in exon 3.

HLA-G, a non-classical class I molecule of the human MHC Ib family, exhibits limited protein polymorphism but marked diversity at the DNA level. As of the IPD-IMGT/HLA Database Release 3.62 (October 2025), a total of 194 alleles encoding 61 proteins have been documented (Robinson et al., 2024; Barker et al., 2023). Here, we report the identification of a novel allele, HLA-G*01:66, which differs from HLA-G*01:01:22:01 by a single nucleotide change in exon 3. The novel allele was identified during a population genetic study designed to characterize HLA diversity in healthy Chinese volunteers. HLA typing was performed as part of this broad screening effort; the two carriers were not selected based on any clinical or familial criteria.

Genomic DNA was extracted from peripheral blood using the iPure DNA HS kit (IGE Biotechnology, Guangzhou, China). HLA typing across 12 loci (HLA-A, -B, -C, -DRB1, -DRB3/4/5, -DQA1, -DQB1, -DPA1, -DPB1, etc.) was performed via long-range multiplex PCR using ApexHF HS DNA Polymerase (Accurate Biology, Changsha, China). Routine typing was conducted on the PolyseqOne nanopore platform (Polyseq Inc., Beijing, China), and novel alleles were confirmed using Oxford Nanopore Technologies (ONT, Oxford, United Kingdom). PolyseqOne libraries were prepared using PY-DTB101/102 and PY-BLP101 kits (Polyseq Inc., Beijing, China), sequenced on PY-NFC001 flow cells for 12 h, and base-called with Kant v1.0.1 in high-accuracy mode (420 bp/s). Fastq files from both platforms were error-corrected with NanoFix-AI. HLA genotyping was then performed with NanoHLA- Resolve Assign v1.0.5 (DAFEI Biotech, Guangzhou, China) against the IPD-IMGT/HLA Database. The error-corrected consensus sequence for HLA-G*01:66 from the two platforms was identical across the full 7.1 kb, with no nucleotide discrepancies.

The novel allele was submitted to GenBank (Accession No. PX126642) and the IPD-IMGT/HLA Database (HWS10101356). It was officially named HLA-G*01:66 by the WHO Nomenclature Committee in September 2025, following current naming guidelines (Marsh et al., 2010). HLA-G*01:66 is a novel allele differing from HLA-G*01:01:22:01 by a single nucleotide change at position 508 in exon 3 (A>G). This mutation converts codon 146 from AAG to GAG, thereby substituting the encoded lysine with glutamic acid (K146E) (Figure 1). The potential functional impact of this substitution remains to be determined.

**FIGURE 1:**
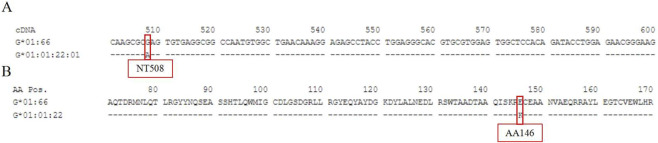
Note: The red box in **(A)** indicates that HLA‐G*01:66 is a novel allele differing from HLA‐G*01:01:22:01 by a single nucleotide change at position 508 in exon 3 (A>G); the red box in **(B)** indicates that this mutation converts codon 146 from AAG to GAG, thereby substituting the encoded lysine with glutamic acid (K146E).

Of the 163 healthy individuals genotyped (corresponding to 326 alleles), two were found to be heterozygous for the novel HLA-G*01:66 allele. The other HLA-G alleles in these carriers were HLA-G*01:01:04 and HLA-G*01:01:01. Despite shared similarities at other HLA loci, the two individuals were confirmed to be unrelated. Their detailed HLA genotypes are presented in Table 1. The screening yielded an observed allele frequency of 0.61% for the HLA-G*01:66 allele. We note that the true population frequency may differ, as our sample size offers limited precision for this estimate.

**TABLE 1:** The HLA genotypes of two individuals with HLA-G*01:66.

Sample number	HLA-A	HLA-B	HLA-C	HLA-DRB1	HLA-DRB3	HLA-DQA1	HLA-DQB1	HLA-DPA1	HLA-DPB1	HLA-E	HLA-F	HLA-G
DF02101	*33:03:01	*51:01:02	*14:02:01	*14:04:01	*02:02:01	*01:01:01	*05:03:01	*02:02:02	*03:01:01	*01:03:01	*01:01:01	*01:04:01
	*74:02:01	*58:01:01	*03:02:02	*01:01:01	—	*01:04:01	*05:01:01	*01:03:01	*02:01:02	*01:01:01	*01:01:02	*01:66
DF00829	*02:07:01	*51:01:02	*01:02:01	*14:54:01	*02:02:01	*01:04:01	*05:02:01	*02:02:02	*31:01:01	*01:03:01	*01:01:01	*01:01:01
	*74:02:01	*46:01:01	*14:02:01	*14:04:01	*02:02:01	*01:04:01	*05:03:01	*02:02:02	*05:01:01	*01:03:02	*01:01:01	*01:66

Note: “ * ”represents a separator, and after the separator are different alleles of the same gene.

## Data Availability

The original contributions presented in this study are included in the article and its supplementary material; further inquiries can be directed to the corresponding author. The novel HLA-G allele sequence data are publicly available. This data can be found at GenBank under the accession number PX126642.
